# Supplementary figures and images for: Linking spatial patterns of terrestrial herbivore community structure to trophic interactions
Source: eLife. 2019 Oct 2;8:e44937. doi: 10.7554/eLife.44937 (PMC6805123; doi:10.7554/eLife.44937)

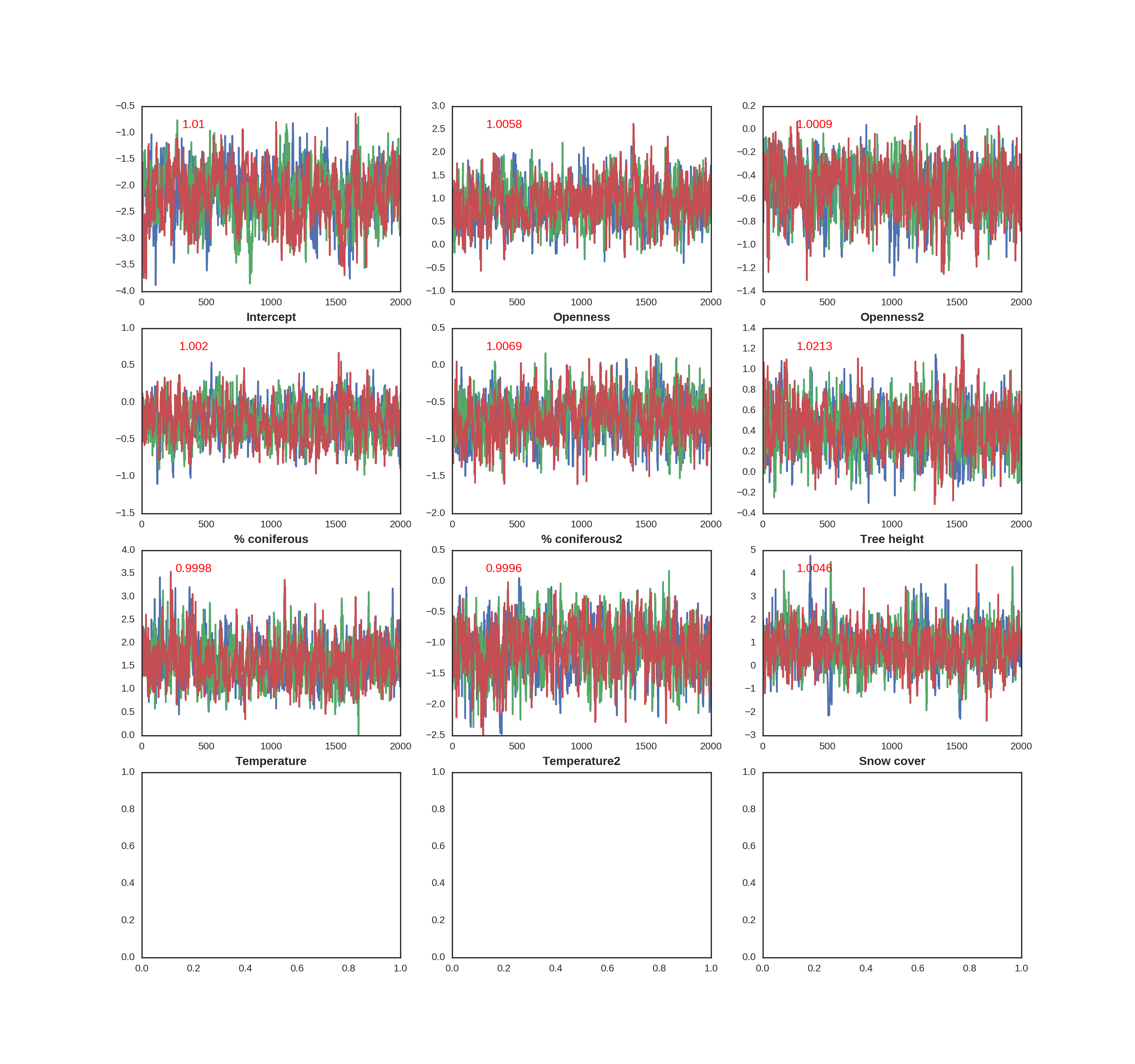

Supplement: Supplementary file 1. [file elife-44937-supp1.zip › Eurasian_Elk_gamma_traces.png]

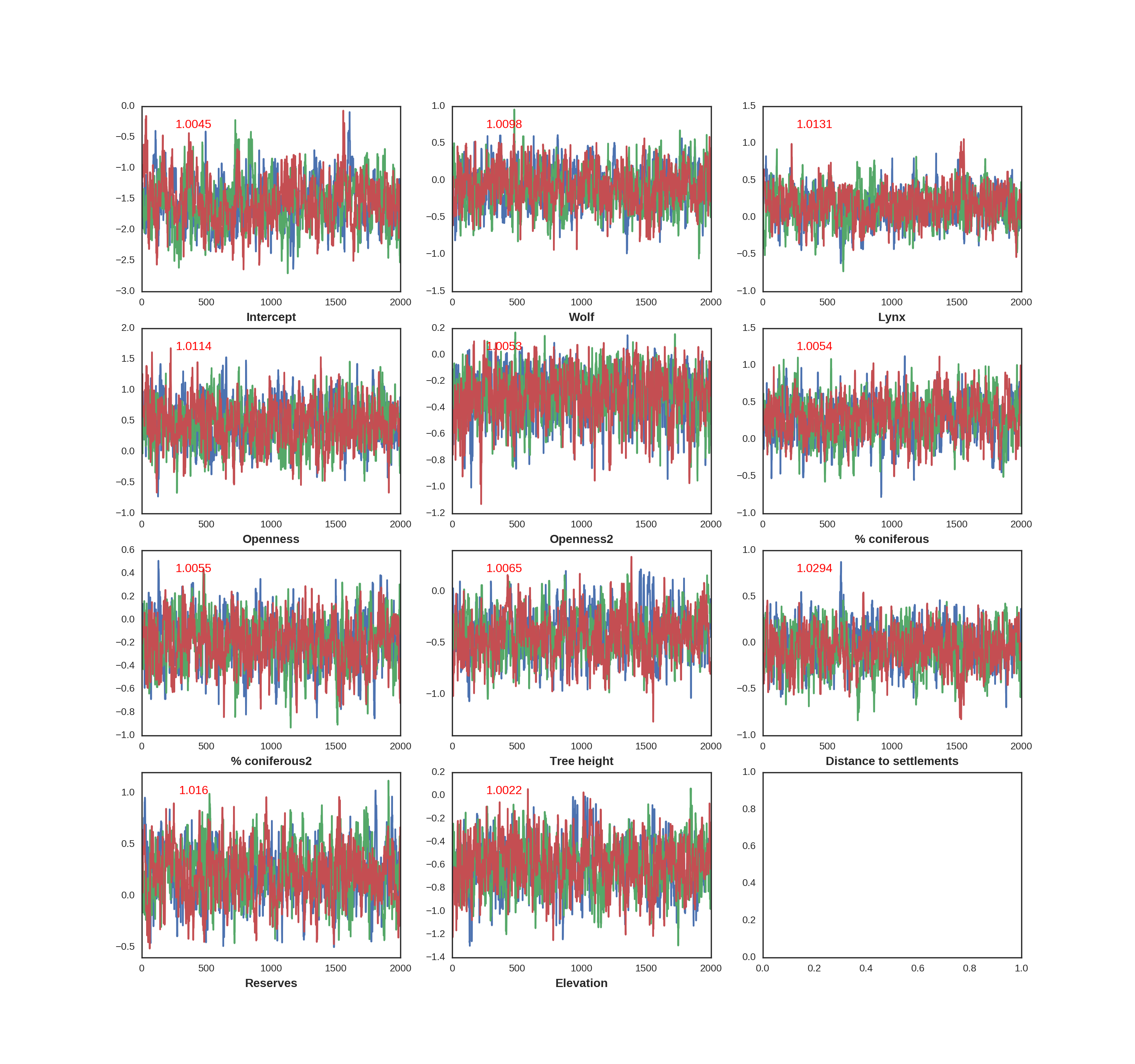

Supplement: Supplementary file 1. [file elife-44937-supp1.zip › Eurasian_Elk_lambda_traces.png]

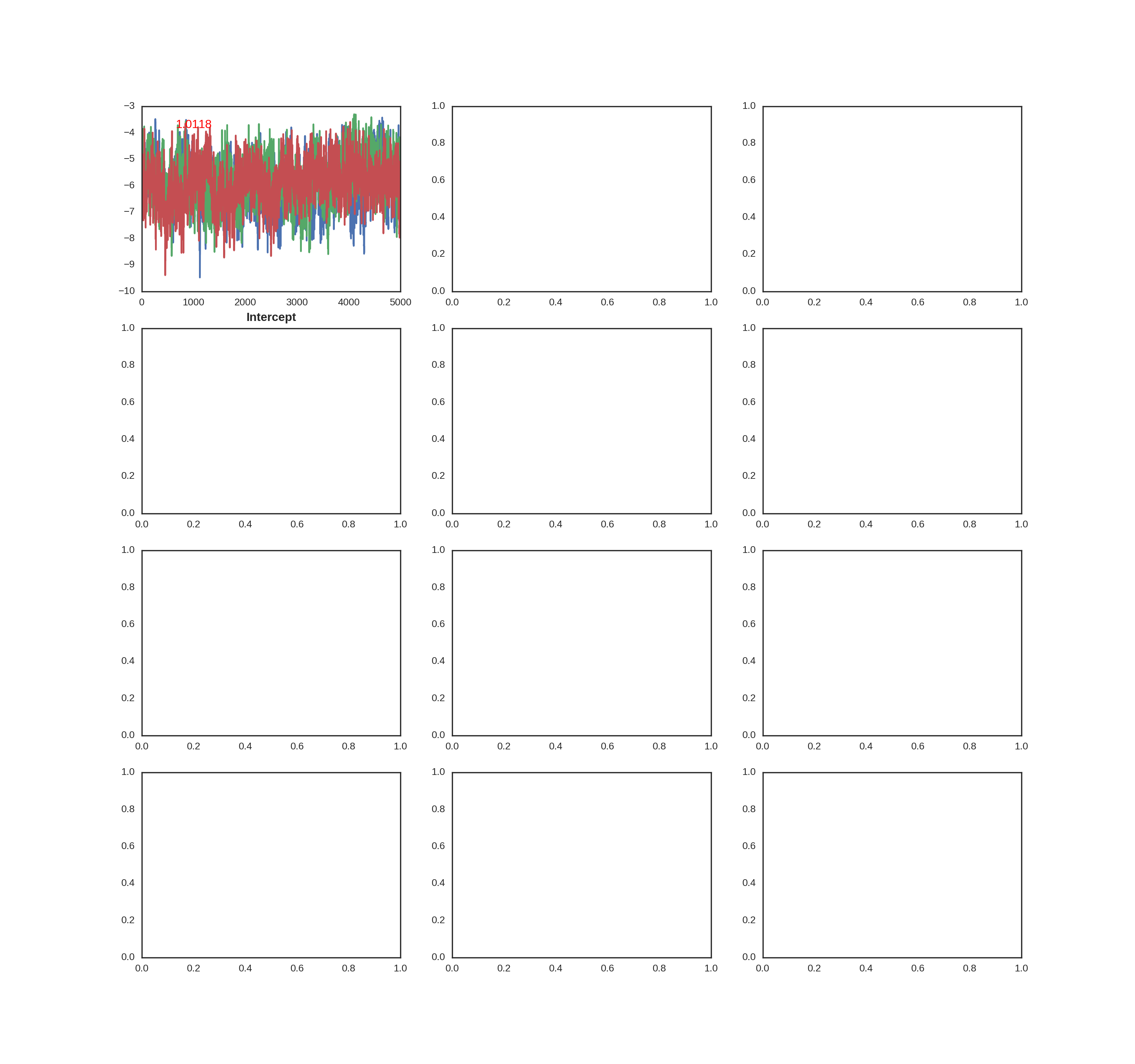

Supplement: Supplementary file 1. [file elife-44937-supp1.zip › Eurasian_Lynx_gamma_traces.png]

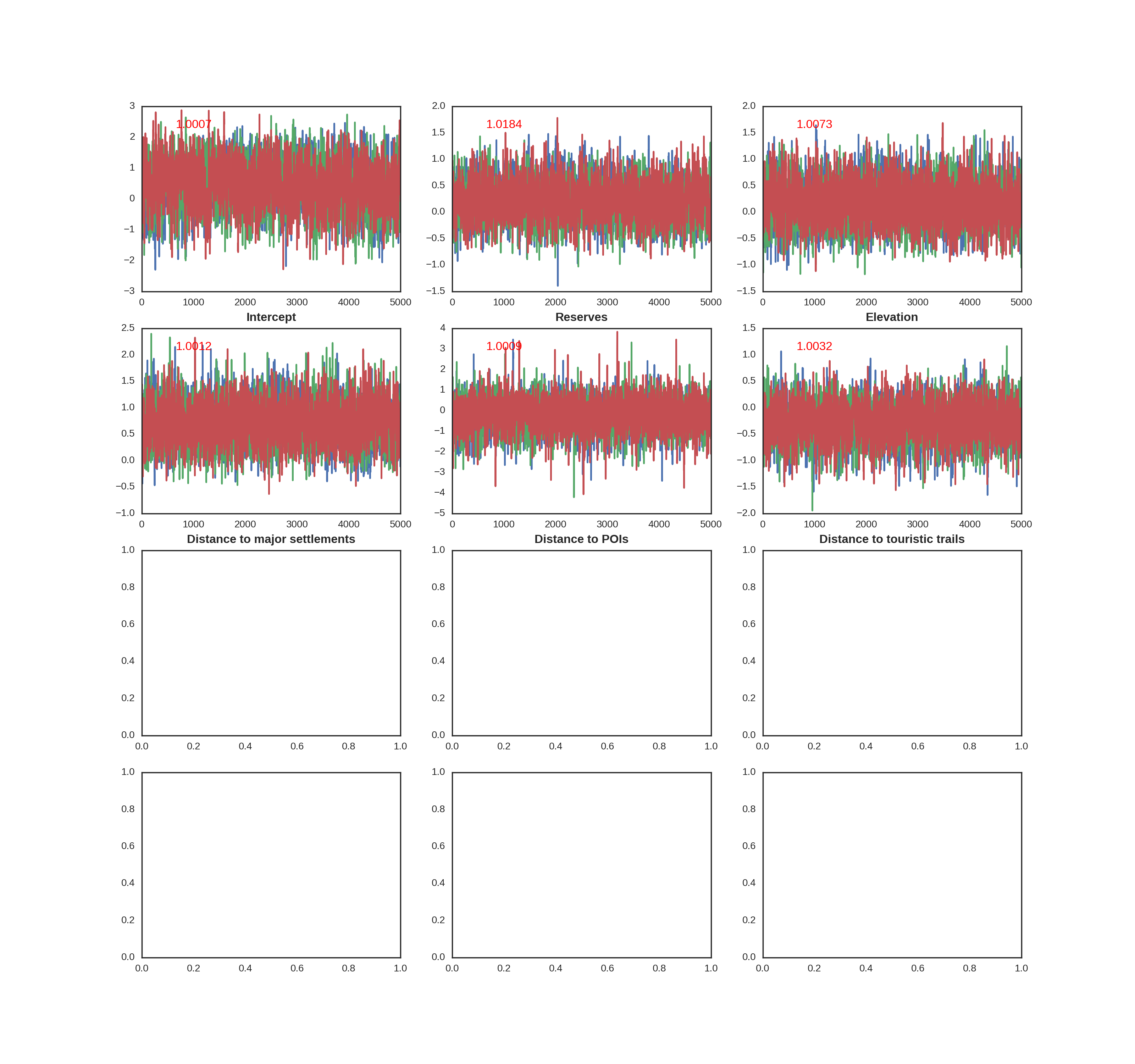

Supplement: Supplementary file 1. [file elife-44937-supp1.zip › Eurasian_Lynx_lambda_traces.png]

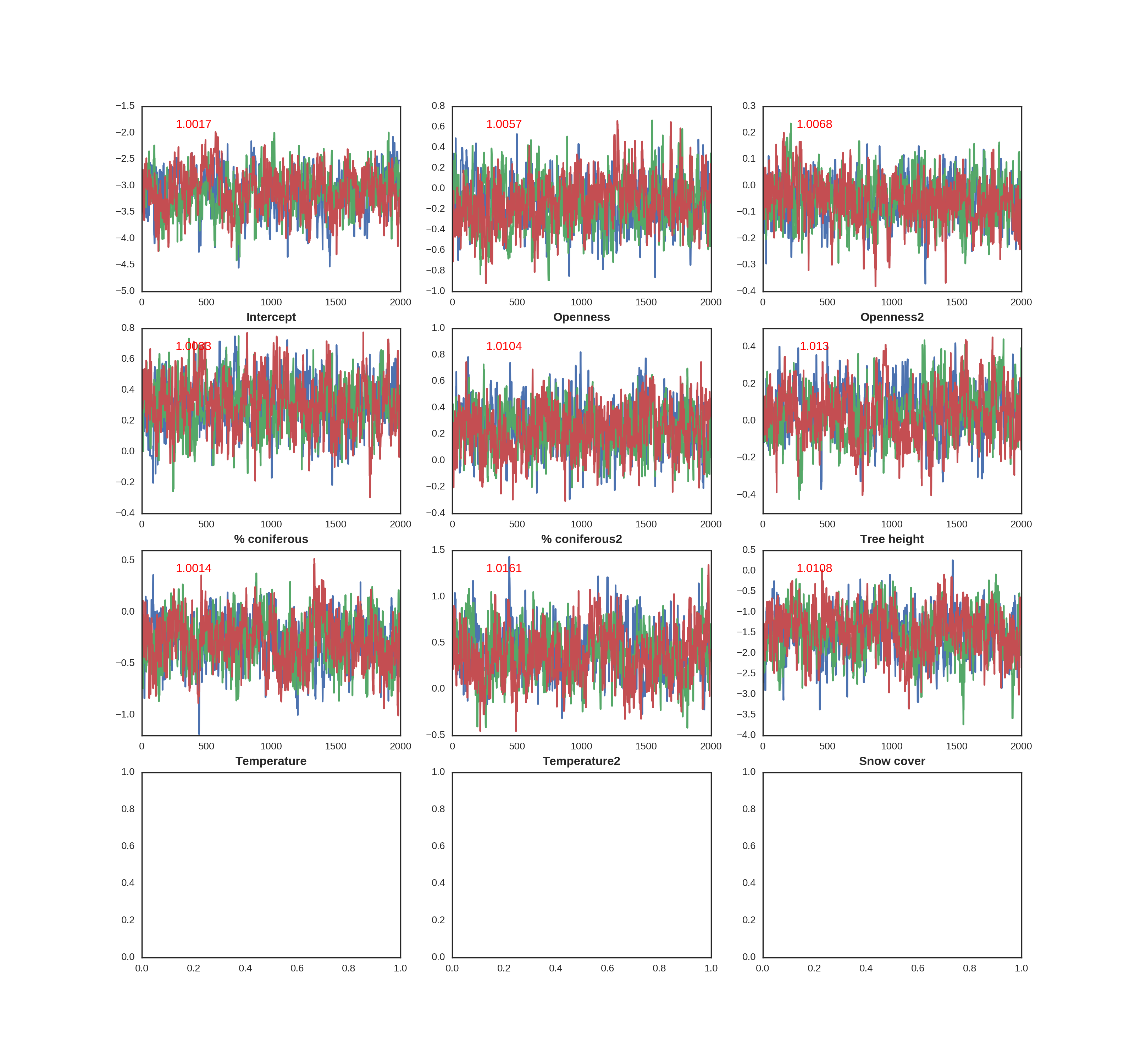

Supplement: Supplementary file 1. [file elife-44937-supp1.zip › European_Bison_gamma_traces.png]

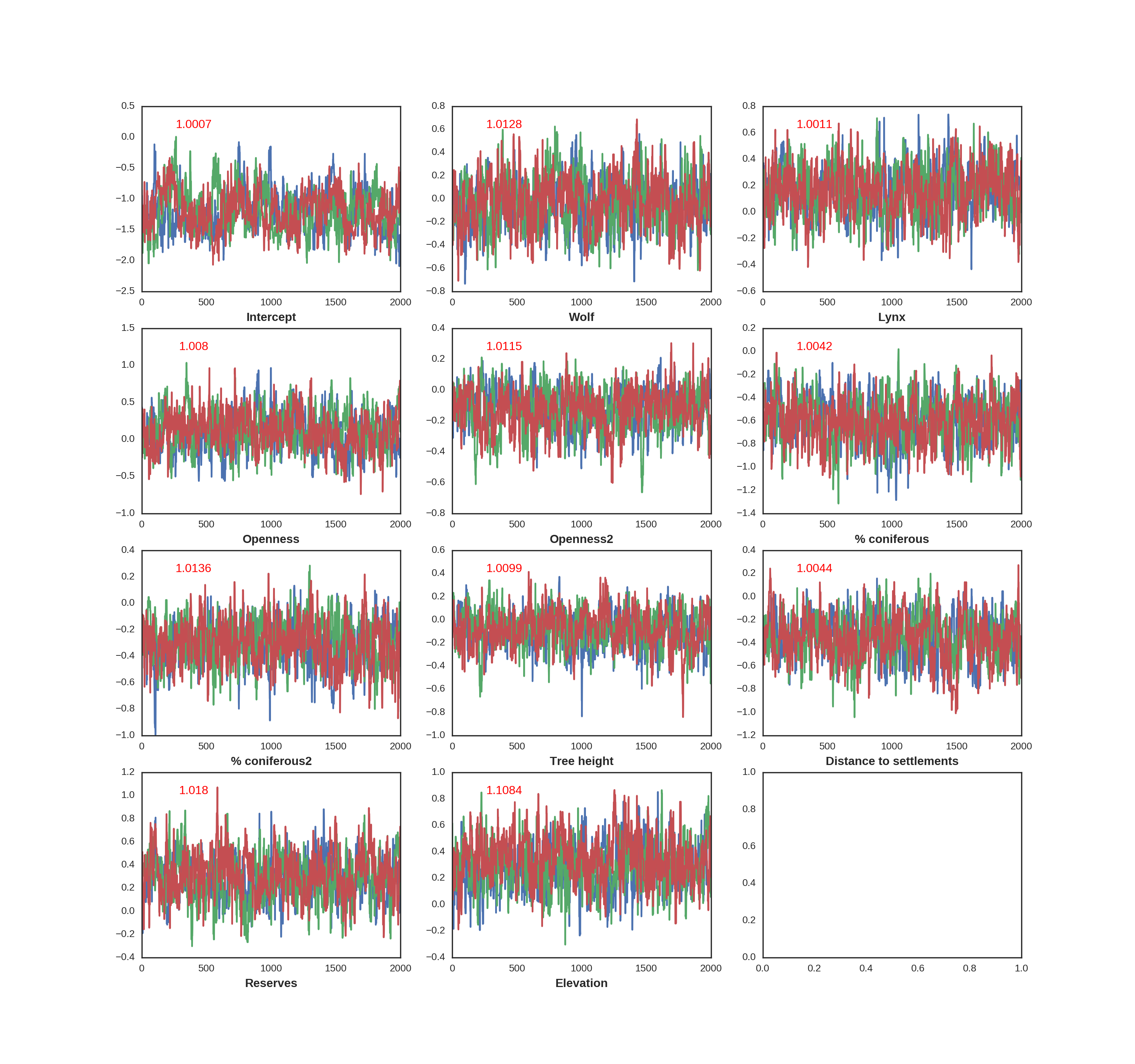

Supplement: Supplementary file 1. [file elife-44937-supp1.zip › European_Bison_lambda_traces.png]

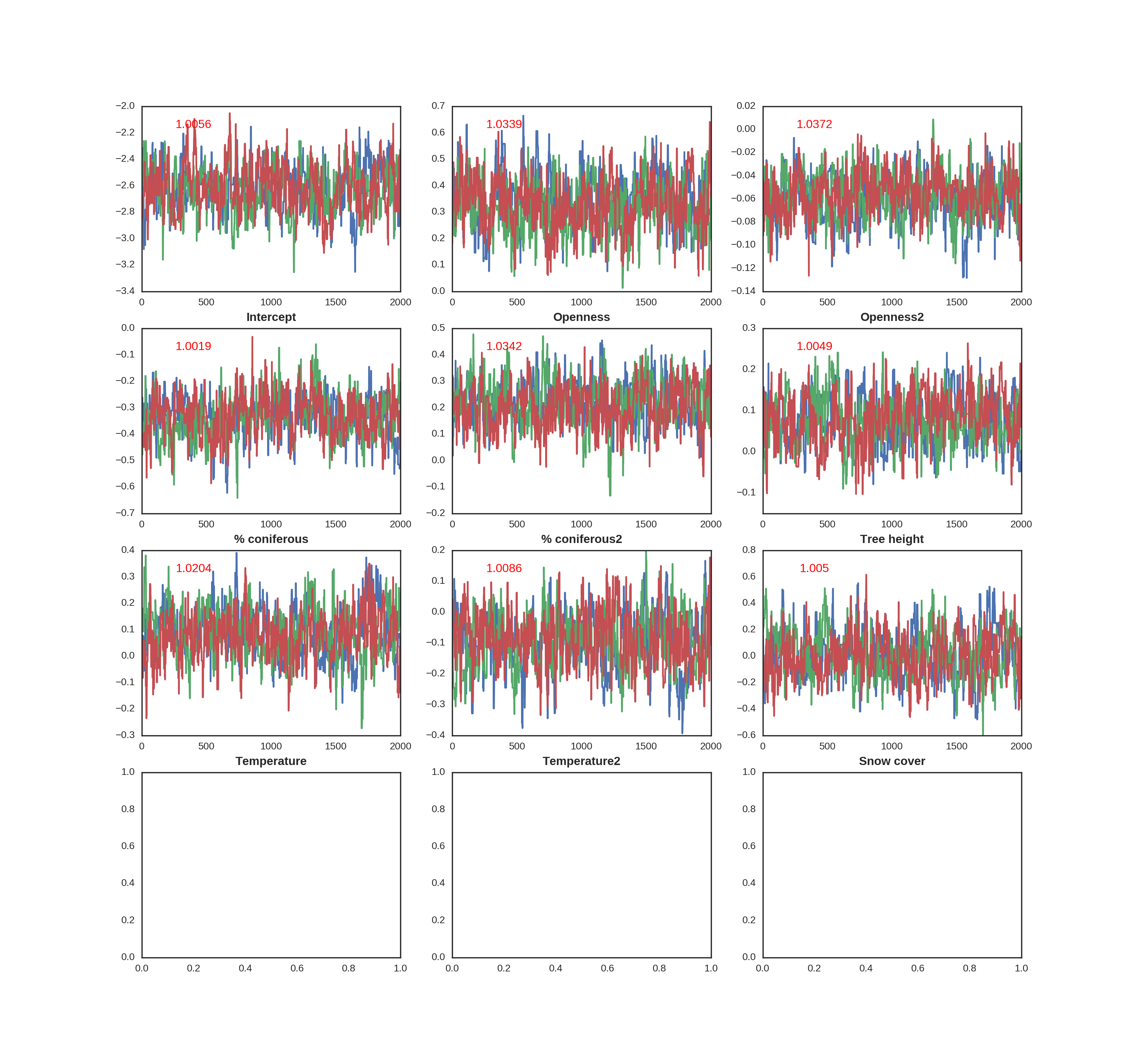

Supplement: Supplementary file 1. [file elife-44937-supp1.zip › Red_Deer_Female_gamma_traces.png]

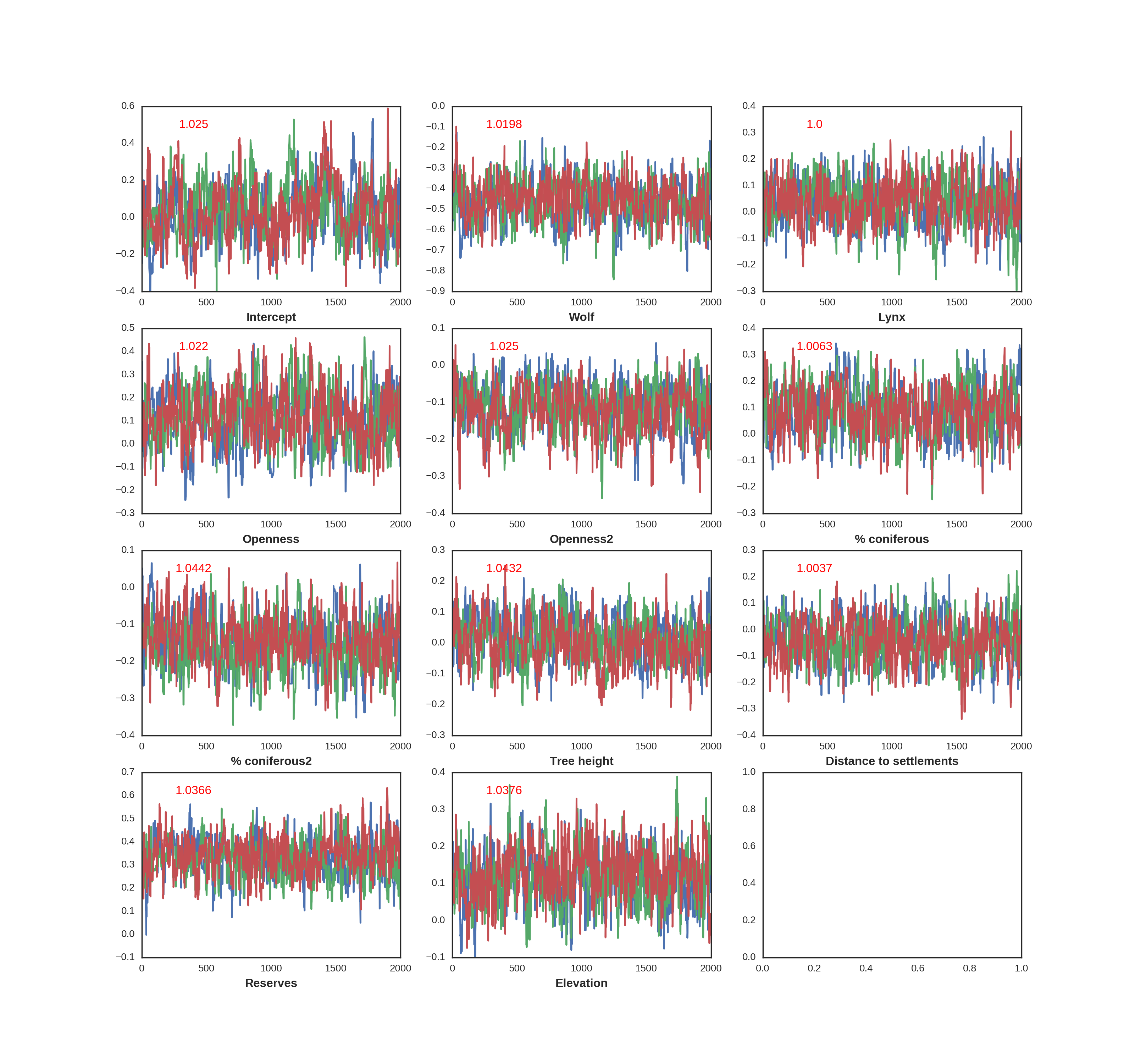

Supplement: Supplementary file 1. [file elife-44937-supp1.zip › Red_Deer_Female_lambda_traces.png]

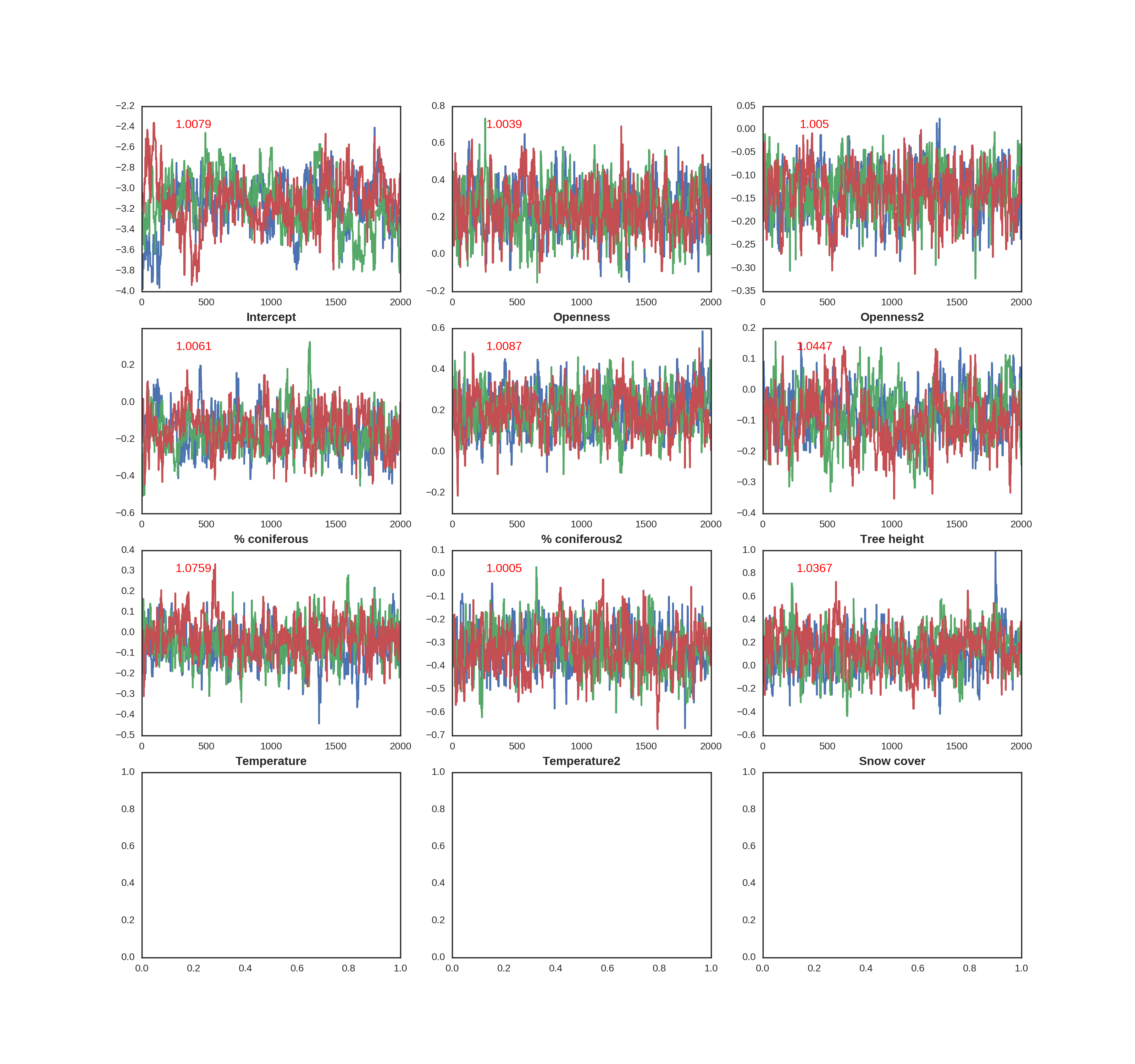

Supplement: Supplementary file 1. [file elife-44937-supp1.zip › Red_Deer_Male_gamma_traces.png]

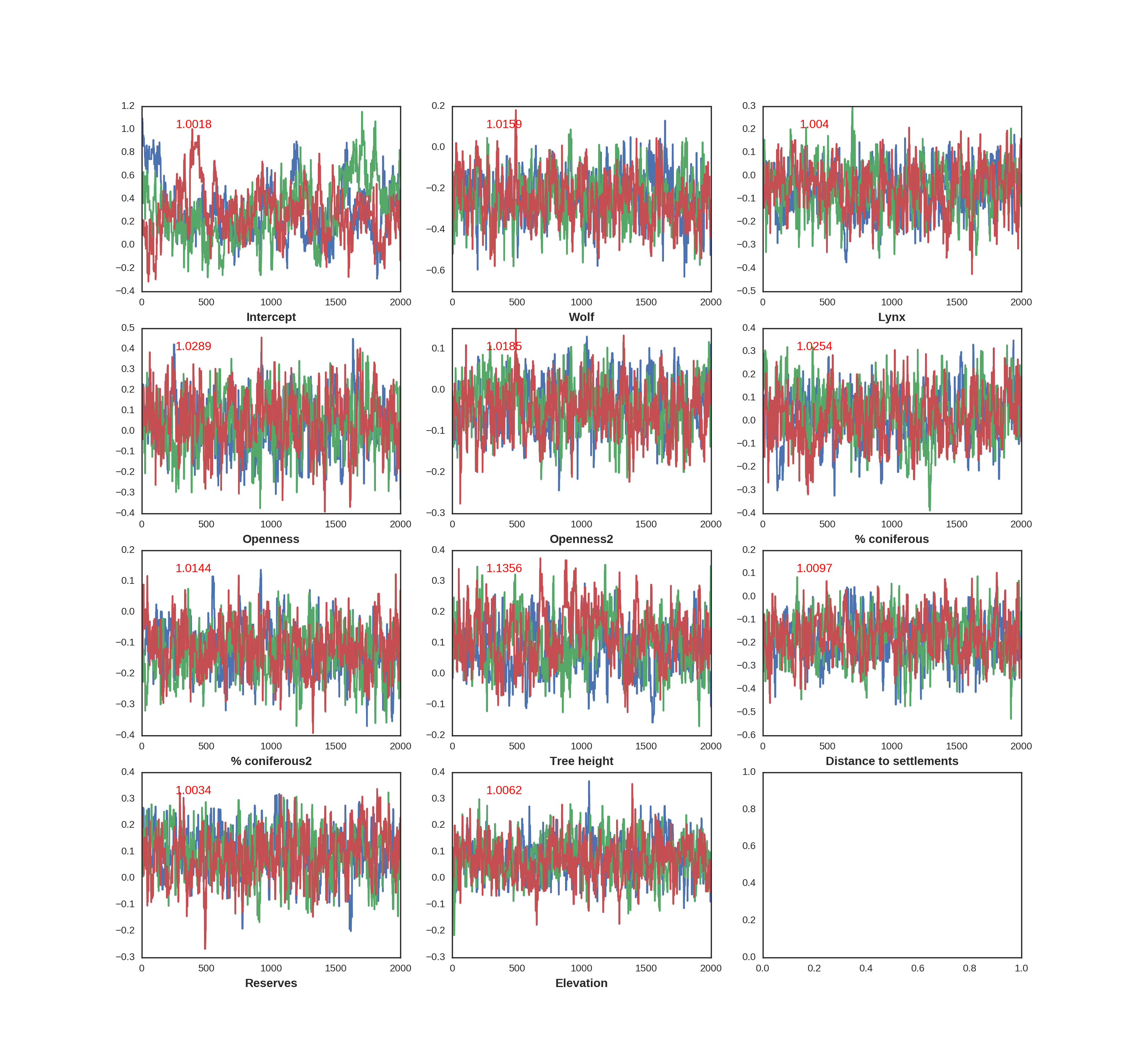

Supplement: Supplementary file 1. [file elife-44937-supp1.zip › Red_Deer_Male_lambda_traces.png]

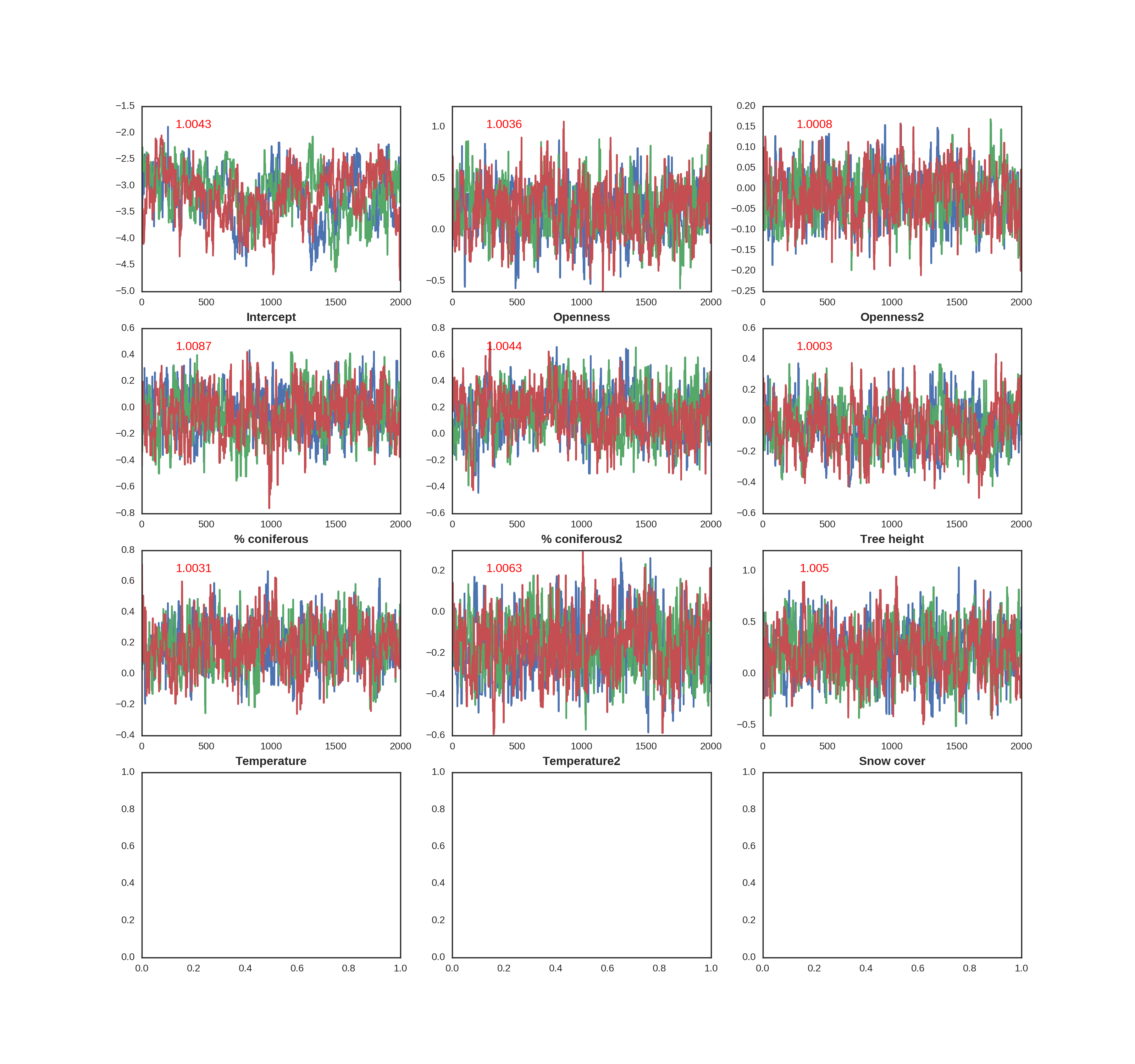

Supplement: Supplementary file 1. [file elife-44937-supp1.zip › Roe_Deer_gamma_traces.png]

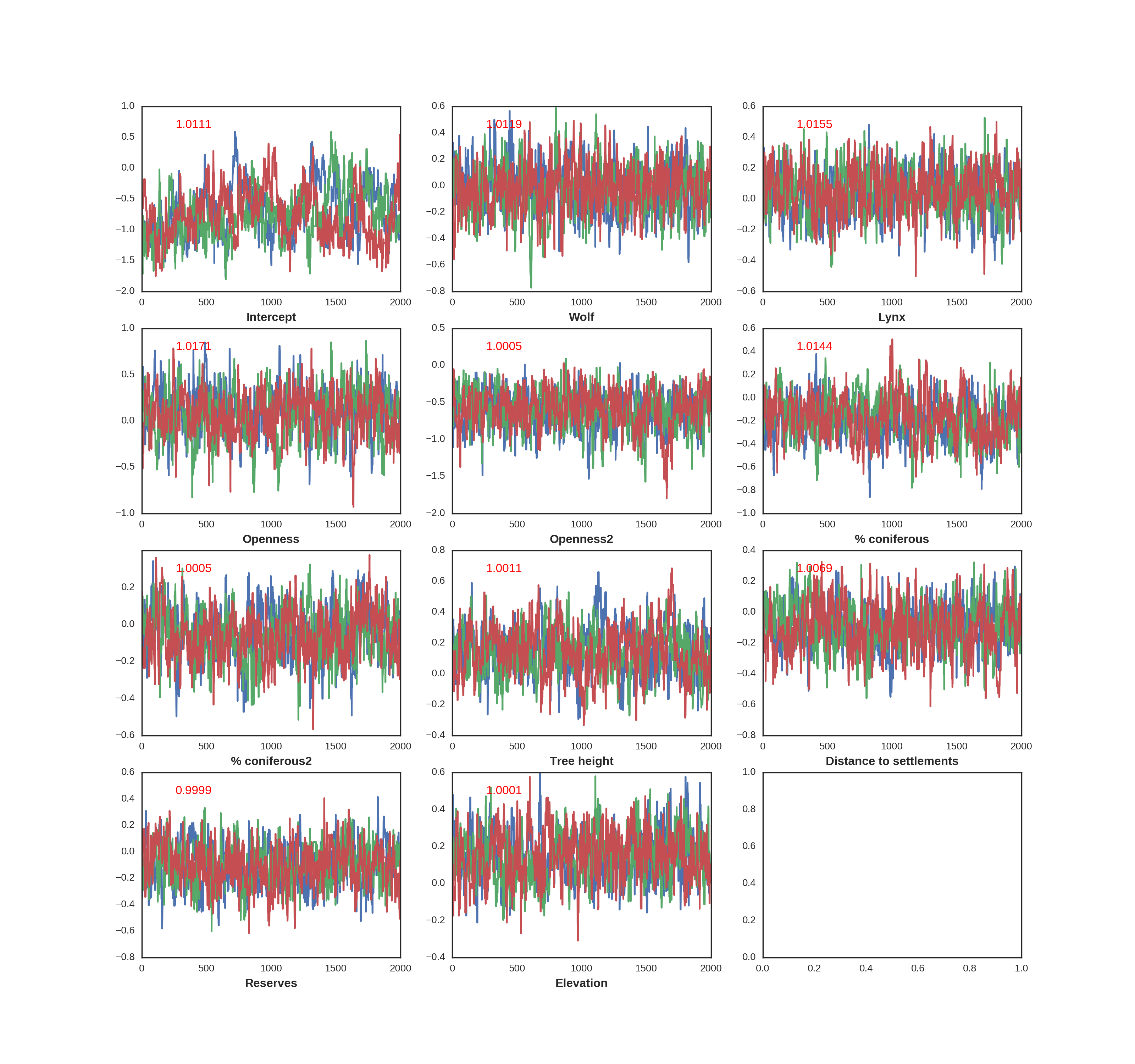

Supplement: Supplementary file 1. [file elife-44937-supp1.zip › Roe_Deer_lambda_traces.png]

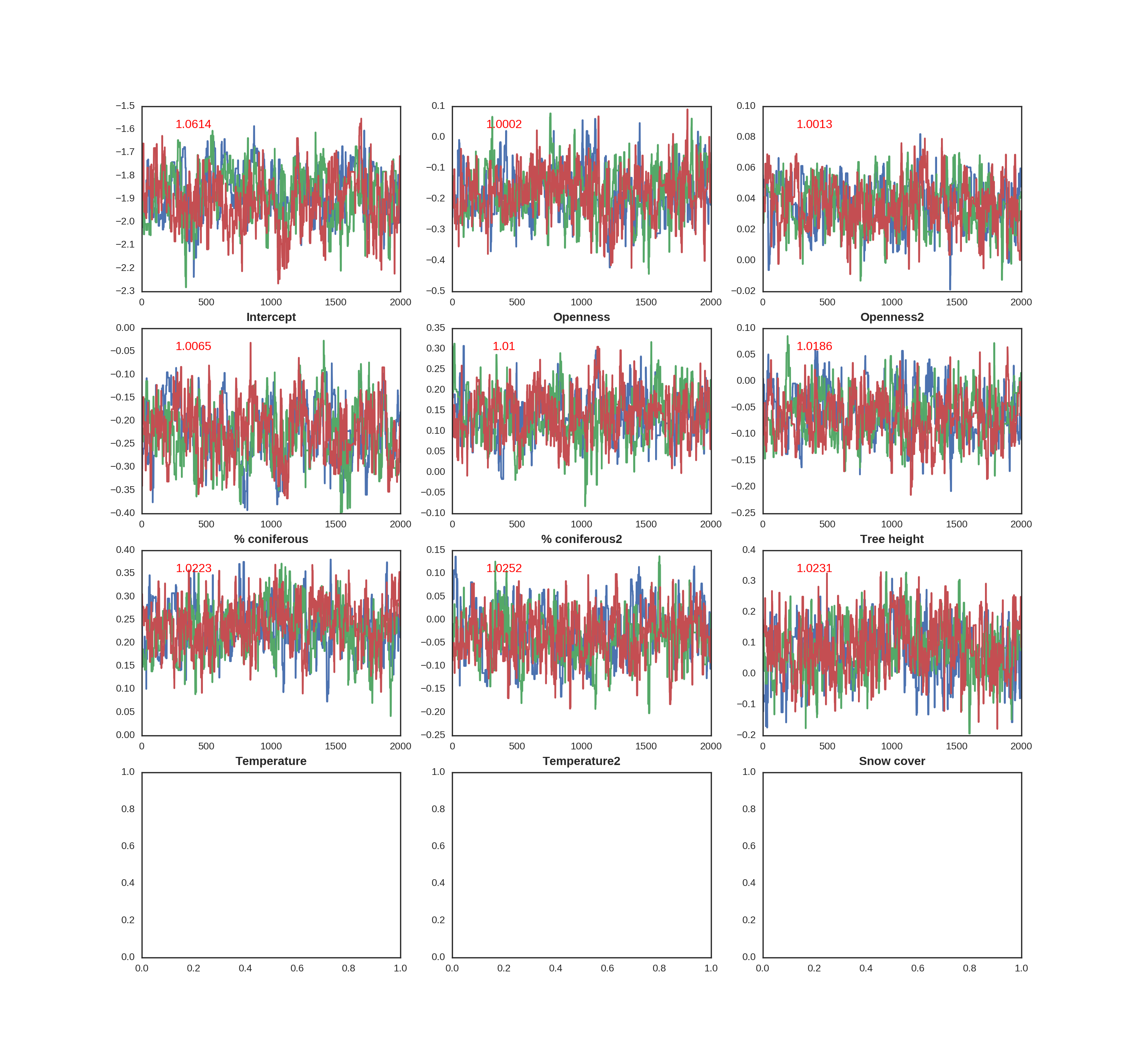

Supplement: Supplementary file 1. [file elife-44937-supp1.zip › Wild_Boar_gamma_traces.png]

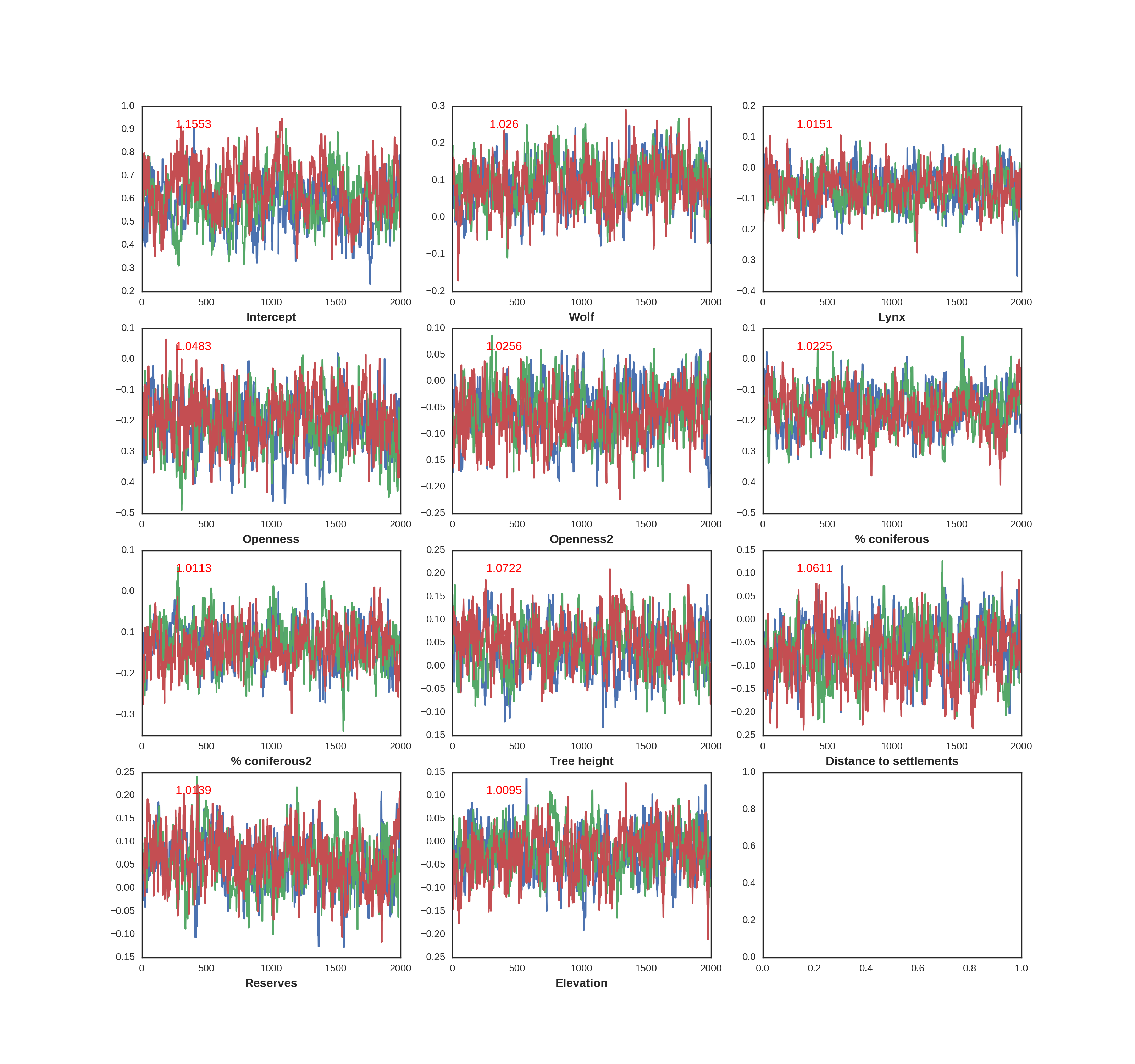

Supplement: Supplementary file 1. [file elife-44937-supp1.zip › Wild_Boar_lambda_traces.png]

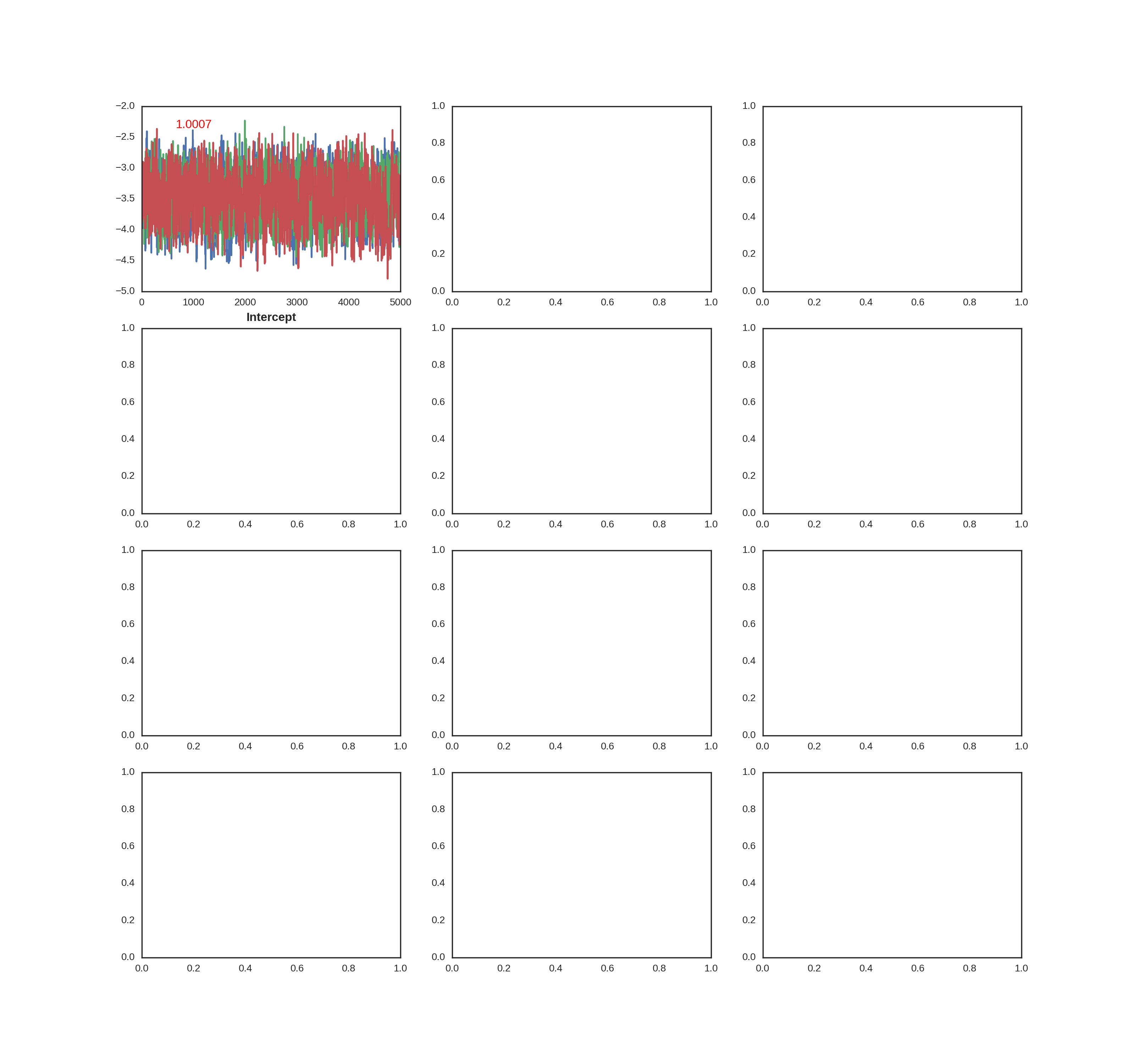

Supplement: Supplementary file 1. [file elife-44937-supp1.zip › Wolf_None_gamma_traces.png]

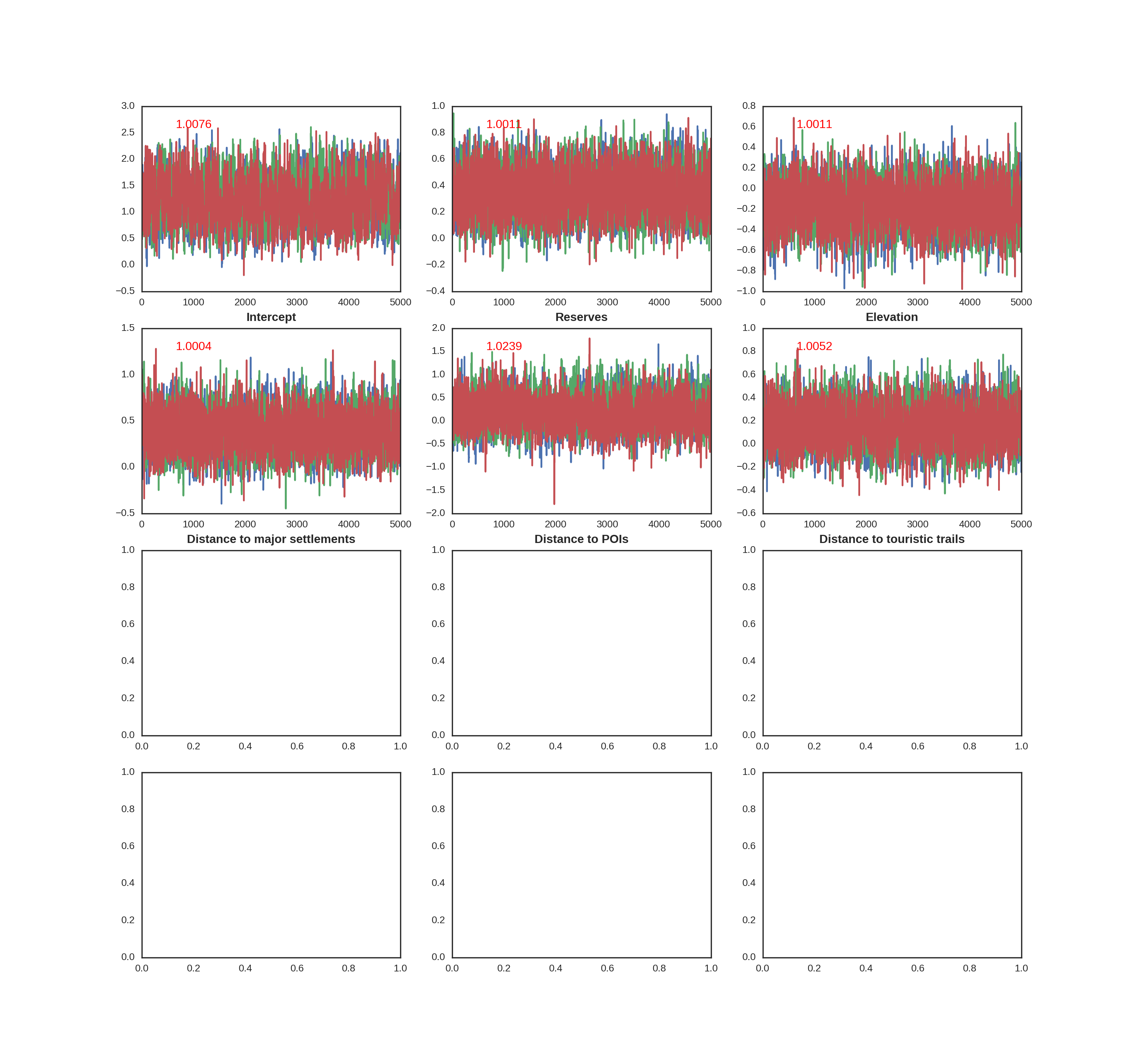

Supplement: Supplementary file 1. [file elife-44937-supp1.zip › Wolf_None_lambda_traces.png]
