# Supplementary material for: Linking spatial patterns of terrestrial herbivore community structure to trophic interactions
Source: eLife. 2019 Oct 2;8:e44937. doi: 10.7554/eLife.44937 (PMC6805123; doi:10.7554/eLife.44937)
Supplement: Transparent reporting form [file elife-44937-transrepform.docx]

***eLife’s* transparent reporting form**

We encourage authors to provide detailed information *within their submission* to facilitate the interpretation and replication of experiments. Authors can upload supporting documentation to indicate the use of appropriate reporting guidelines for health-related research (see EQUATOR Network), life science research (see the [BioSharing Information Resource](https://biosharing.org/)), or the [ARRIVE guidelines](http://www.plosbiology.org/article/info:doi/10.1371/journal.pbio.1000412) for reporting work involving animal research. Where applicable, authors should refer to any relevant reporting standards documents in this form.

If you have any questions, please consult our Journal Policies and/or contact us: [editorial@elifesciences.org](mailto:editorial@elifesciences.org).

**Sample-size estimation**

- You should state whether an appropriate sample size was computed when the study was being designed
- You should state the statistical method of sample size computation and any required assumptions
- If no explicit power analysis was used, you should describe how you decided what sample (replicate) size (number) to use

Please outline where this information can be found within the submission (e.g., sections or figure legends), or explain why this information doesn’t apply to your submission:

*No explicit power analysis was used prior to our field data collection using camera traps. As our study was of an observational, rather than experimental nature, and as we investigated complex spatial interactions between many species representing multiple trophic levels, we were unable (to our knowledge) to run an explicit power analysis. However, in our methods we ensured a large enough number of sampled locations to successfully fit our complex spatial models (diagnostics are presented in the supplementary file Appendix S1). Our sampling methodology, together with a short discussion of all the arbitrary decisions we had to take, are described in detail in the main text in the Methods section.*

**Replicates**

- You should report how often each experiment was performed
- You should include a definition of biological versus technical replication
- The data obtained should be provided and sufficient information should be provided to indicate the number of independent biological and/or technical replicates
- If you encountered any outliers, you should describe how these were handled
- Criteria for exclusion/inclusion of data should be clearly stated
- High-throughput sequence data should be uploaded before submission, with a private link for reviewers provided (these are available from both GEO and ArrayExpress)

Please outline where this information can be found within the submission (e.g., sections or figure legends), or explain why this information doesn’t apply to your submission:

*This information does not apply to our submission as our study was not of an experimental nature.*

- Report exact p-values wherever possible alongside the summary statistics and 95% confidence intervals. These should be reported for all key questions and not only when the p-value is less than 0.05.

Please outline where this information can be found within the submission (e.g., sections or figure legends), or explain why this information doesn’t apply to your submission:

*The statistical approach used in our study is thoroughly described in the Methods section, including descriptive and formal definition of our spatial models. We do not report the p-values as we used Bayesian approach as our inference framework.*

**Group allocation**

- Indicate how samples were allocated into experimental groups (in the case of clinical studies, please specify allocation to treatment method); if randomization was used, please also state if restricted randomization was applied
- Indicate if masking was used during group allocation, data collection and/or data analysis

Please outline where this information can be found within the submission (e.g., sections or figure legends), or explain why this information doesn’t apply to your submission:

*This information does not apply to our submission as our study was not of an experimental nature.*

- We encourage you to upload relevant additional data files, such as numerical data that are represented as a graph in a figure, or as a summary table
- Where provided, these should be in the most useful format, and they can be uploaded as “Source data” files linked to a main figure or table
- Include model definition files including the full list of parameters used
- Include code used for data analysis (e.g., R, MatLab)
- Avoid stating that data files are “available upon request”

Please indicate the figures or tables for which source data files have been provided:

*The source code of our analyses together with the source data files can be found in our github repository: https://github.com/mripasteam/herbiscapes/.*
